# Automated White Blood Cell Counting in Nailfold Capillary Using Deep Learning Segmentation and Video Stabilization

**DOI:** 10.3390/s20247101

**Published:** 2020-12-11

**Authors:** Byeonghwi Kim, Yuli-Sun Hariyani, Young-Ho Cho, Cheolsoo Park

**Affiliations:** 1 Department of Computer Engineering, Kwangwoon University, Seoul 01897, Korea; 354651@kw.ac.kr (B.K.); yulisun@telkomuniversity.ac.id (Y.-S.H.); 2 School of Applied Science, Telkom University, Bandung 40257, Indonesia; 3 Department of Electrical Communication, Daelim University, Anyang-Si 13916, Korea

**Keywords:** deep learning, image registration, semantic segmentation, video stabilization, white blood cell counting

## Abstract

White blood cells (WBCs) are essential components of the immune system in the human body. Various invasive and noninvasive methods to monitor the condition of the WBCs have been developed. Among them, a noninvasive method exploits an optical characteristic of WBCs in a nailfold capillary image, as they appear as visual gaps. This method is inexpensive and could possibly be implemented on a portable device. However, recent studies on this method use a manual or semimanual image segmentation, which depends on recognizable features and the intervention of experts, hindering its scalability and applicability. We address and solve this problem with proposing an automated method for detecting and counting WBCs that appear as visual gaps on nailfold capillary images. The proposed method consists of an automatic capillary segmentation method using deep learning, video stabilization, and WBC event detection algorithms. Performances of the three segmentation algorithms (manual, conventional, and deep learning) with/without video stabilization were benchmarks. Experimental results demonstrate that the proposed method improves the performance of the WBC event counting and outperforms conventional approaches.

## 1. Introduction

White blood cells (WBCs), also called leukocytes, are important components of the immune system in the human body, since their deficiency could cause various health conditions, such as sepsis [1], infectious diseases [2,3], and cancer [4]. The WBC monitoring usually requires the extraction of a blood sample by an experienced medical staff using specialized equipment. Patients who need continuous monitoring of the WBCs daily, for conditions such as neutropenia, have drawbacks due the equipment’s inefficiency and inconvenience. To improve the WBC monitoring method, various noninvasive techniques based on optical methods have been proposed. One method assesses the vessel in the oral mucosa [5], and another one is based on optical characteristics in nailfold capillaries, where WBCs are recognized as visual gaps or particles [6]. This study focuses on the nailfold capillary images because it could implement the WBC monitoring system while improving both patient comfort and measurement accessibility.

**Figure 1:**
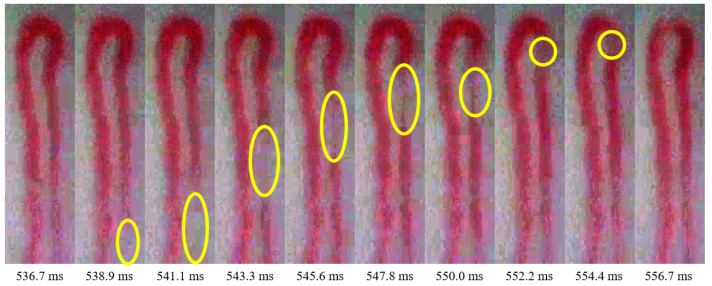
Example of a WBC event (black ellipse) over a sequence of nailfold capillary images.

When the diameter of a WBC reaches that of the capillary through which it flows, the blood flow along that capillary is interrupted by the WBC, causing a gap in a capillary image. A sequence of images with gaps allows for seeing the WBCs flowing through the capillary, as illustrated in Figure 1, where each gap is considered as a WBC event. Using the visual gap characteristic, Bourquard et al. [7] proposed a semi-automated pipeline for gap numeration to achieve the noninvasive WBC analysis using a portable and low-cost capillaroscope and an image processing method, namely, spatiotemporal representation and the Radon transform. However, they conducted the capillary segmentation process in a semi-manual way. As manual processing is substantially slower than a computerized processing, it represents a bottleneck for automated analysis. To prevent this, an automated image segmentation approach based on a deep learning algorithm could be a solution, since the convolutional neural networks have fostered efficiency in computer vision tasks, including object recognition [8,9], detection [10,11], and segmentation [12,13,14,15]. For instance, the U-Net has been widely utilized for image segmentation [16,17] given its high performance and efficient use of GPU memory [18]. Likewise, we also adopt the U-Net [13] for our semantic segmentation problem aiming to automate capillary identification.

Additionally, inaccurate capillary detection and miss-detection of the WBC event might occur as a small finger motion is magnified when seen through a microscope. To mitigate this type of artifact, we apply motion compensation while also adopting an efficient version of image registration for translations [19] that reduces the capillary motion and accumulates data in case of a large unwanted movement in the recorded capillary videos [20]. The main contribution of this study is the automation of WBC event counting using a deep learning approach supported by video stabilization for robustness against motion artifacts. The proposed method consists of an automatic capillary segmentation method using deep learning, video stabilization, and WBC event detection algorithms.

The remainder of this paper is organized as follows: In Section 2, related works about semantic segmentation and video stabilization are presented; details of the proposed method are explained in Section 3; Section 4 provides the results of the experiment settings and results; Section 5 provides the discussion; lastly, Section 6 gives the summary and conclusions of this work.

## 2. Related Work

### 2.1. Semantic Segmentation

Image segmentation aims to extract useful regions from an image as a set of contours or sub-images for subsequent analysis and interpretation. To extract regions of interest, thresholding approaches [21,22] can be used to determine pixel intensities that discard the background. In addition, clustering approaches, such as the *K*-means [23], group similar pixel intensities over a region. However, these approaches are sensitive to the image characteristics and scenes, such as shaded images and the presence of multicolored objects, thus undermining their performance.

Since the introduction of convolutional neural networks [24], the consistency of visual recognition has substantially improved in many visual tasks, including classification [8,9,25,26], detection [10,11,27], and segmentation [12,28,29,30,31]. Such deep learning approaches leverage high-level features of input images, providing robustness against noise compared to conventional methods. Deep learning using convolutional neural networks has been exploited in various applications of medical image processing including segmentation [13,14,15].

We adopt u-net architecture for capillary segmentation because it uses global location and context information simultaneously, and works well with few training samples [13]. The u-net can be trained using the few data images in end-to-end manner, where the whole image in the forward pass can directly produce segmentation maps in order to retain the full context of the input images [13].

### 2.2. Video Stabilization

Camera motion while recording video causes the captured objects to move accordingly. When unintended, such motion can cause noise such as image blurring, which undermines the quality and consistency of the recorded image. To prevent this problem, stabilization improves the quality of video by eliminating unintended movements, including translations and rotations. Various approaches are available to remove motion artifacts from video. As the proposed method relies on video recorded by a human operator, the video is likely to include unwanted movements. Moreover, noise is amplified in the video because the capillaroscopy device magnifies the capillaries during recording. Therefore, video stabilization becomes essential for processing and analyzing capillary videos.

We only assume device translations during video recording [32]. Therefore, video stabilization in the proposed method should determine translation vectors. Given that these vectors contain the directions and magnitudes of camera movements, it is possible to fix the capillary positions by shifting the frames based on the vectors along the opposite direction. To determine the translation vector between two consecutive images, we adopt a computationally efficient version of an image registration method [19]. Specifically, given two images, Guizar-Sicairos et al. [19] determine the translation vector that maximizes the cross-correlation between them. To this end, each image is expressed in the frequency domain by applying the fast Fourier transform. Then, elementwise multiplication is performed between the converted images, and then the result is reverted back into the spatial domain. For image registration, capillary labels corresponding to frames are exploited rather than raw frames to mitigate the impact of noise.

If large camera movements occur, image registration may fail in the worst case. The magnification in capillary videos of small hand motions can produce such large camera movements, representing a potentially severe problem in the proposed method. A previous study [20] has addressed this problem by accumulating movements. Likewise, we adopt the same approach to reduce the extent of large movements.

## 3. Proposed WBC Counting Method

**Figure 2:**
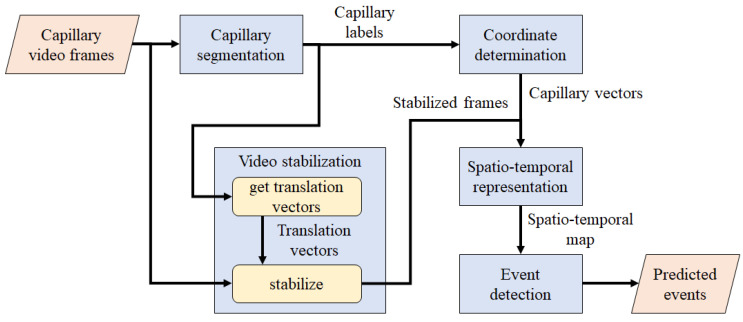
Overview of the proposed WBC counting process.

The proposed WBC counting method is illustrated in Figure 2, where the capillary video and predicted events are its input and output, respectively. During frame extraction, the input video is preprocessed to extract regions of interest containing capillaries for the subsequent analysis. Capillary segmentation extracts the capillaries and removes the background from the video. Then, coordinate determination selects the coordinates from the labels of each capillary to extract the corresponding pixel intensities. Video stabilization is applied using image registration based on the translation vectors acquired from the capillary labels. A spatiotemporal representation transforms the intensities acquired from the selected coordinates of each capillary in a frame into a 1D array, and the set of arrays obtained from all the frames are represented as a 2D matrix, whose *x*- and *y*-axes represent time and the corresponding intensity array, respectively. Finally, event detection predicts the events in the spatiotemporal map using the Radon transform and local maxima detection. Below, we detail each step of the proposed method.

### 3.1. Frame Extraction

**Figure 3:**
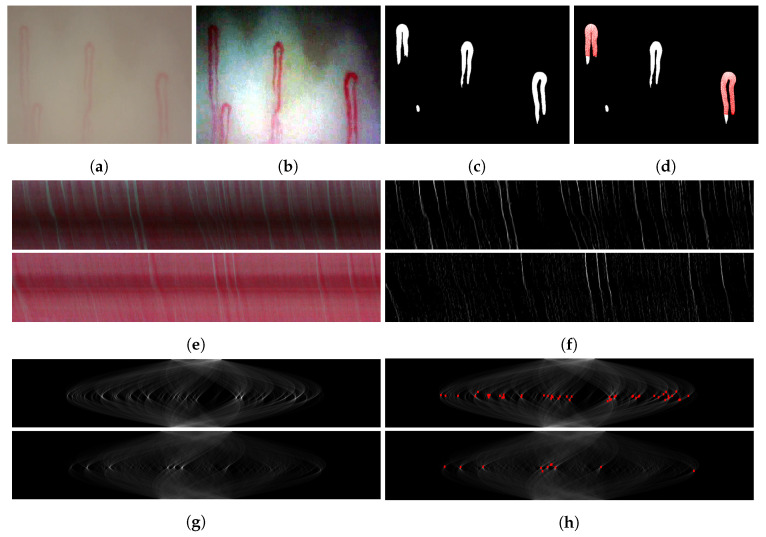

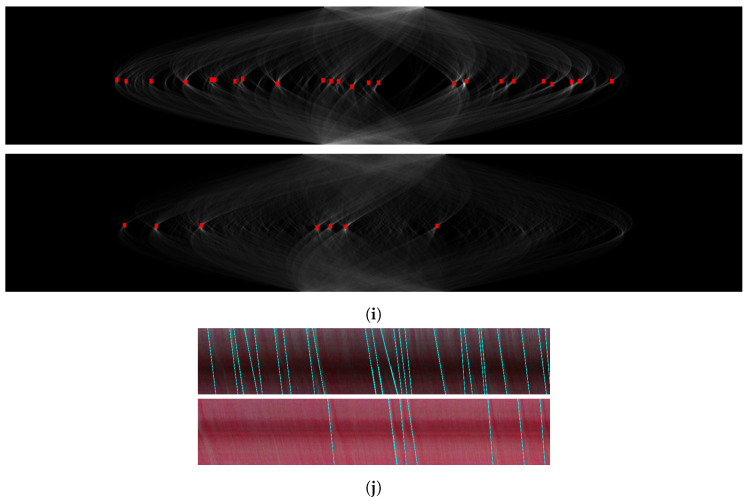
Application of the proposed WBC event counting method. (**a**) raw frame; (**b**) processed frame; (**c**) labels of capillaries; (**d**) coordinates of labels; (**e**) ST maps from capillaries; (**f**) processed ST maps; (**g**) polar maps obtained by applying Radon transform; (**h**) detected local maxima from (**g**); (**i**) local maxima selected as base events; (**j**) reconstructed lines according to events in (**i**).

In this step, all the frames are extracted from an input video. The capillaries in raw images are hardly distinguishable from the background (see Figure 3a), which results in poor capillary segmentation and event counting due to their low-quality information. To enhance the contrast for better visibility of capillaries in a video, we apply histogram equalization to the red, green, and blue channels in the frames (see Figure 3b).

### 3.2. Capillary Segmentation

To extract the representative information from the capillaries captured in a video, the capillary labels should be determined to characterize their locations and appearances. To this end, we adopt deep learning segmentation and compare its performance with a conventional segmentation method.

#### 3.2.1. Deep Learning-Based Segmentation

For a deep learning-based segmentation of the capillaries captured in a video, we adopt the semantic segmentation model introduced in [13]. Given a frame image (see Figure 3b), the model outputs pixelwise capillary labels (see Figure 3c), which determine the locations and shapes of the capillaries. Specifically, an RGB image is fed to the model encoder and compressed into a dense representation as a multidimensional vector through consecutive convolutional and pooling layers. Then, the model decoder up-samples the compressed representation through consecutive convolutional and up-sampling layers.

**Figure 4:**
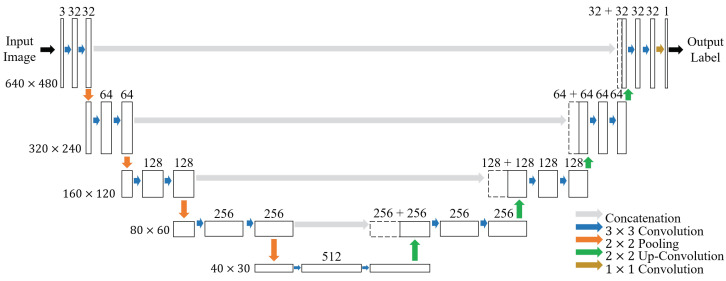
Proposed deep learning model for capillary segmentation.

Figure 4 describes the model architecture of the proposed deep learning segmentation. Each box represents a feature map with dimension [*c*, *w*, *h*], where *c* is the channel size (a value on top of the box) and *w* and *h* are the width and height (values on the side of the box) of the feature map, respectively. Each colored arrow denotes the corresponding operation (see the figure legends) between the connected feature maps. A gray arrow indicates concatenation, which is followed by a 2 × 2 up-convolution represented as a green arrow. An orange arrow represents a 2 × 2 max-pooling layer to reduce the spatial size followed by a 3 × 3 convolutional layer represented as a blue arrow. At the end of the model, the dark yellow arrow indicates a 1 × 1 convolution to map each multidimensional channel onto a scalar, such that the feature map is converted into a single-channel map, which contains the pixelwise labels of the capillaries in the input image.

We use a dataset containing 1358 capillary images and the corresponding ground-truth labels for training and validating the model. We divide this dataset into 950 and 408 images for the training and validation sets, respectively. After training the model for 100 epochs with Adam optimization on binary cross-entropy loss, learning rate of 0.001, and batch size of 3, 91.11% validation accuracy was obtained regarding the mean intersection over union.

#### 3.2.2. Conventional Segmentation

As a benchmark test, a conventional segmentation algorithm is implemented based on the capillary optical characteristics. Since a capillary appears as a red region such as Figure 3b, the color information of the region could be utilized for the segmentation process. Therefore, pixels in a capillary area contain less green and blue components than those from the background. For the segmentation, capillaries are labelled by subtracting the sum of the green and blue components from the weighted red component in an image to achieve a relatively large contrast with respect to the background. In addition, the subtraction results are squared to highlight the intensities of the capillary and discard those of the background, whose intensity is low in general. The conventional segmentation algorithm for an images is implemented as follows:

(1) $$Label=ReLU{\left(\lambda I_{R}-\left(I_{G}+I_{B}\right)\right)}^{2}$$

(2) ReLU(x)=max(0,x)

where λ is the weight of the red component and $I_{R}$, $I_{G}$, and $I_{B}$ denote the intensities of the red, green, and blue components of an image. Weight λ (1.5 in this study) compensates for the intensity of the red component by the summation of the green and blue components.

### 3.3. Video Stabilization

**Figure 5:**
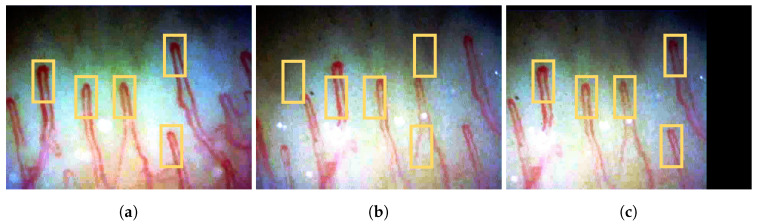
Example of the video stabilization process: (**a**) the first frame set as a reference, (**b**) a frame before the stabilization, and (**c**) a frame stabilized based on the reference. The yellow rectangles indicate the initial position of capillaries in the reference frame. Note the shift of the frame after the stabilization process.

To minimize unwanted motion artifacts in a capillary video stream, the translation vectors of video frames corresponding to a reference frame are determined [20]. A motion artifact is measured between two frames; current frame and reference frame. The video stabilization process estimates how much a frame has moved from the reference frame, where the amount and direction of the movement are represented as a translation vector. By adding the translation vectors to the frames, the capillary positions could be corrected to keep the initial position as the reference, which is usually the first frame [32]. Figure 5a–c illustrate an example of the video stabilization process. Considering the first frame in Figure 5a as the reference, the frame in Figure 5b is aligned to the position of the reference frame. The stabilized frame in Figure 5c shows the aligned capillaries to the reference locations.

However, it is empirically observed that the video stabilization process shows poor performance when the amount of the translation becomes large. As the amount of the translation has more chances to become large as time goes given a fixed reference frame, there would be more chances for the video stabilization process not to show the best performance if the first frame is consistently used as the reference frame. In the sense, a simple yet effective way to keep the amount of the translation small would be to periodically update the reference frame every *p* frames (*p* is set as 50), not to fix the reference frame to the first frame. It could alleviate the performance degradation issue because the periodic update shortens the time interval between frames to be stabilized and a reference frame and therefore the amount of the translation could be kept small.

When a reference frame is updated, subsequent frames are stabilized with respect to the next reference frame. However, the video stabilization process translates the positions of capillaries in the subsequent frames to the position of those in not the first frame but the next reference frame. To stabilize the subsequent frames with respect to the first frame, the video stabilization process also keeps a history of the translations among all reference frames. When the translation between the first and the last frames is equivalent to the sum of all translation vectors, a single variable, denoted by “reference” in Algorithm 1, would be enough to represent the history.

It is empirically observed that a capillary video is better stabilized with the translation vectors acquired from not raw frames but the labels obtained from the segmentation process detailed in Section 3.2. For the reason, the video stabilization process stabilizes all capillary videos based on the translation vectors from the labels.

In addition, the video stabilization algorithm is also applied to the labels. Even though the capillary segmentation could generate such a great quality of capillary masks, it would be much better to utilize multiple capillary masks as an ensemble for more robust masks. However, the multiple capillary masks include capillaries in different positions across time. To keep all capillaries of the masks in the same position, the same video stabilization process is applied to the masks.

Algorithm 1 describes the video stabilization process, where “get_translation_vec” returns a translation vector between a reference and an image, and “apply_translation” translates the image based on the translation vector.
**Algorithm 1:** Pseudo code for video stabilization.
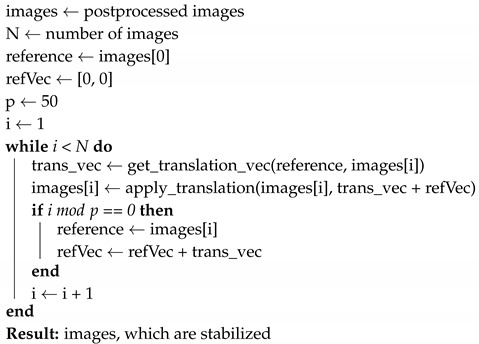


### 3.4. Capillary Coordinate Determination

A label obtained from capillary segmentation includes multiple pixels, but they are not ordered or all of them are not needed for the analysis. Therefore, the important pixels and their orders should be precisely determined. Unlike the method in [7], which applies an interpolation [33] with manual selected information, the automatically selected pixels along the boundary of a capillary with a label are utilized to orderly extract the internal, external, and intermediate coordinates corresponding to the capillary.

**Figure 6:**
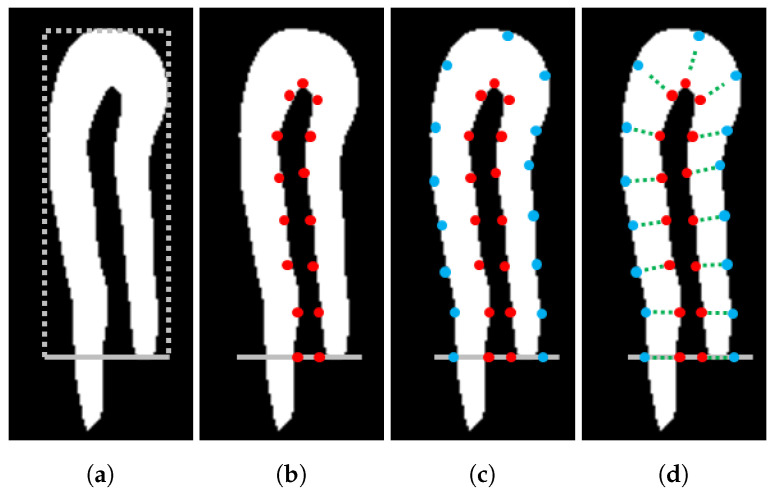
Example of capillary coordinate determination. (**a**) virtual rectangle determining capillary region of interest; (**b**) internal coordinates (red dots); (**c**) external coordinates (blue dots); (**d**) intermediate coordinates (green dots) between pairs of internal and external coordinates.

To determine the coordinates, a virtual rectangle around the labelled capillary is adjusted to establish a region of interest, as illustrated in Figure 6a. The rectangle fully covers the short horizontal side between the two branches of a capillary, and all the coordinates in the internal boundary of each capillary are selected, as illustrated in Figure 6b. Then, an equal number of external coordinates are also chosen, and the external boundary is divided into as many coordinates as the internal coordinates like Figure 6c. This procedure enables the internal coordinates to be paired with the external ones.

Using the internal and external coordinates, the intermediate coordinates are created with the connecting lines across the paired internal and external coordinates, similar to the method in [7], as illustrated in Figure 6d. Using all the determined coordinates, a 1D array is constructed, whose length is the number of the internal coordinates and each element is the average intensity of the corresponding internal, external, and intermediate coordinates. Figure 6 illustrates all the processes of the capillary coordinate determination described in this section, and Figure 3c,d display the results example.

### 3.5. Spatiotemporal Representation

Given a frame, the coordinates of each capillary (Section 3.4) are used to obtain a capillary vector that represents the intensities of the capillary. By concatenating the vectors across all frames over time, we obtain a matrix that represents the intensities of a capillary throughout the video, establishing what we call an ST map. The *x*- and *y*-axes of an ST map indicate the time and capillary vector corresponding to time, respectively. An element of a capillary vector has a high value if it corresponds to the area of the event containing the WBC. As the element of an event moves along the capillary vector over time, the event appears as a line in the ST map. The problem of counting visual gaps is thus a problem of counting lines in an ST map. An example of events appearing in an ST map is shown in Figure 3e.

The raw ST map is sensitive to the quality of the capillary image. Therefore, the ST map should be processed to highlight the event information and eliminate ambient noise. First, we apply a median filter to the ST map to remove weak event information. Then, the unnecessary background is eliminated to highlight the event lines in the ST map. To this end, each row of the ST map is zero-meaned, and the resulting negative values are zeroed. To enhance the contrast between the background after the removal and lines in the ST map, the derivative of the intensities of the lines is calculated while applying the Sobel operator to the processed ST map. As a result, the lines in the ST map become more distinguishable, as illustrated in Figure 3f.

### 3.6. WBC Event Detection

To detect events occurring as lines in an ST map, we adopt a local maxima detection method using the Radon transform, which was introduced in [7]. The Radon transform maps an image given in Cartesian coordinates into an image given in polar coordinates which is called a polar map. The Radon transform is defined as

(3) $$R\left(\theta ,z\right)=\int_{-\infty }^{\infty }f\left(s\sin\theta +z\cos\theta ,-s\cos\theta +z\sin\theta \right)ds$$

where f(i,j) and R(θ,z) are a pixel of a zero-centered image at (i,j) in Cartesian coordinates and a polar map at (θ,z) in polar coordinates, respectively. The map converts lines in an image (ST map) into peaks, as illustrated in Figure 3g. The lines of a processed ST map are thus represented as peaks in the polar map. The peaks in a polar map are illustrated in Figure 3h. By detecting the local maxima in a polar map, the WBC events in a video sequence could be identified.

Given its thickness, a line in an ST map might lead to duplicate detections in the corresponding polar map, as the line is not an ideal one with zero area. To prevent the duplicate detection, some events are eliminated. First, the local maxima whose angle in the polar map contains most events are selected as base events and each local maximum except the base events is removed if its reconstructed line crosses any reconstructed lines of the base events in the ST map. Otherwise, the local maximum is added to the set of base events. The resulting base events are illustrated in Figure 3i, and the reconstructed lines from the base events are shown in Figure 3j.

## 4. Experiments and Results

### 4.1. Experiment Settings

To validate the proposed method, three capillary videos, denoted as videos 1–3, were recorded from three healthy subjects by experts using a portable capillaroscopy device. Each RGB video was recorded for 30 s at 30 frames per second and 640 × 480 resolution. Therefore, the input for the proposed model is a 900 × 480 × 640 × 3 array. Among the capillaries in each video, the best two were selected to ensure a clear shape in the video for segmentation. The WBC events were counted by four experts to establish the ground truth. We obtained the Korean IRB approval (No. P01-201903-11-02) to conduct the experiments involving human participants.

As hyper-parameters for the segmentation methods in Section 3.3, the labels were binarized with empirical thresholds of 150 and 80 for the deep learning and conventional segmentation, respectively. Figure 3b,c respectively show a frame of a video and the averaged label of the outputs using deep learning segmentation across all frames.

Hereinafter, the following abbreviations are used for the methods, that is, the method names with and without ‘S’ indicate a video with and without stabilization. ‘Manual’, ‘Conventional’, and ‘DNN’ denote that the capillary labels in segmentation process are obtained manually, the conventional segmentation, and the semantic segmentation based on a deep learning algorithm, respectively. For the evaluation of the proposed methods, the six combinations of the stabilization and segmentation methods are explored to evaluate the performance of WBC event counting. The configuration of each combination method is showed in Table 1.

### 4.2. Results

**Figure 7:**
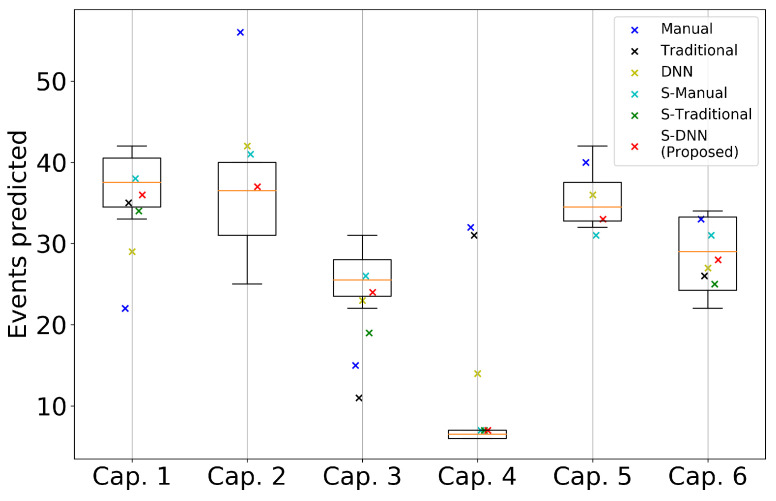
Prediction variability for the capillary videos using all the different segmentation methods. The *x*-axis denotes the index of each capillary from videos 1 to 3, and the *y*-axis the number of predicted events. Each ‘×’ mark indicates the number of predicted events using the corresponding segmentation and video stabilization. The boxplot for each capillary is obtained from the events counted by four experts.

Box-plot in Figure 7 shows the variability of the WBC event counting among the experts and the orange line inside each box indicates its median value, while the different colors of ‘x’ show the number of WBC events predicted by each methods and ‘cap.’ means capillary. In cap. 2 and cap. 5, only four methods were presented because the other two, the Traditional and S-Traditional methods, failed to detect the capillary. It can be seen that our proposed method, denoted by the orange cross-mark, consistently predicts the WBC event number around the median value as the box for each capillary. It shows that the stabilization process and automatic segmentation using DNN improve the performance of predicted events.

**Figure 8:**
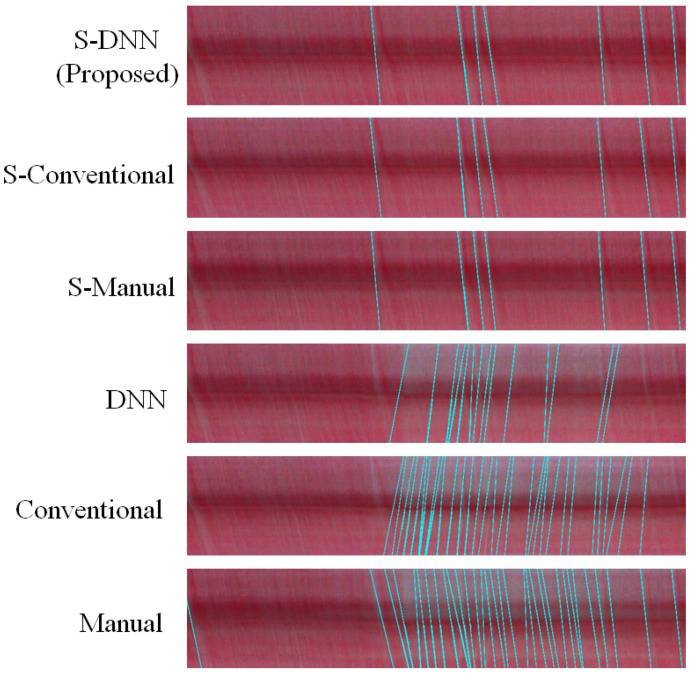
ST maps and reconstructed events (cyan lines) for ST maps detected by event counting for the combinations of video stabilization and capillary segmentation for capillary 4.

**Figure A1:**
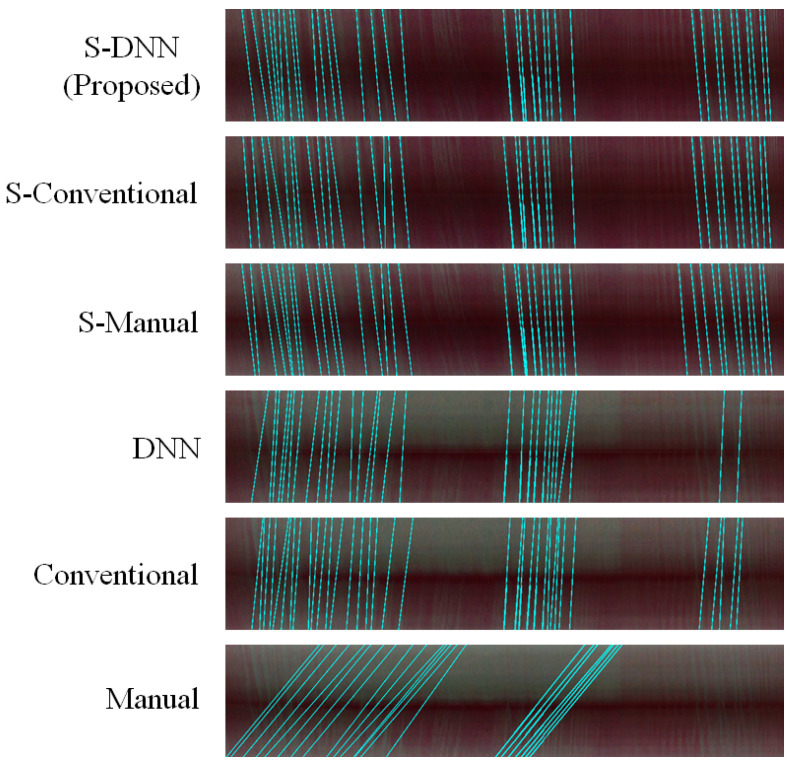
Events reconstructed from event counting using various methods for capillary 1.

**Figure A2:**
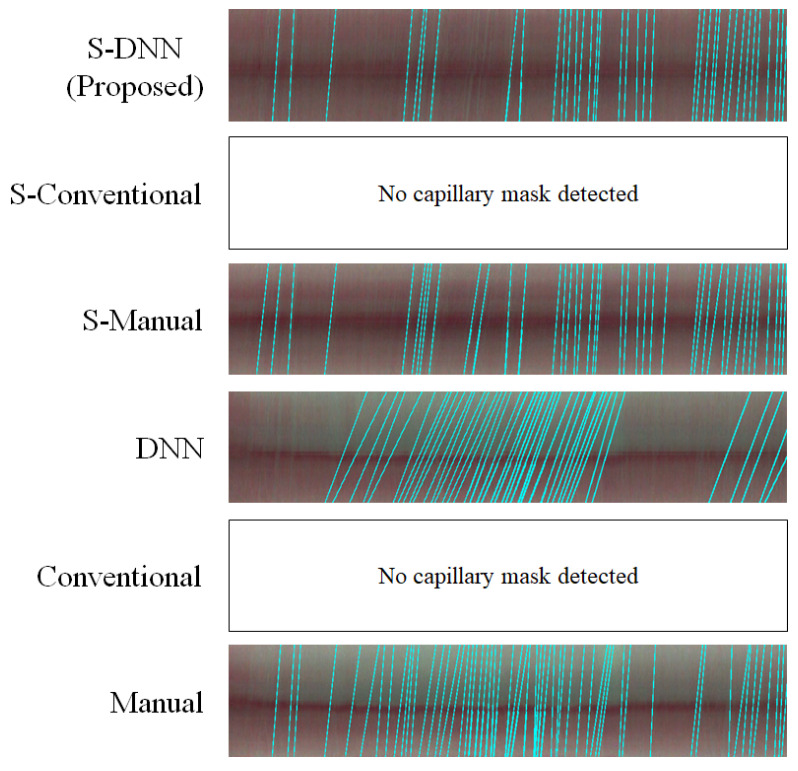
Events reconstructed from event counting using various methods for capillary 2.

**Figure A3:**
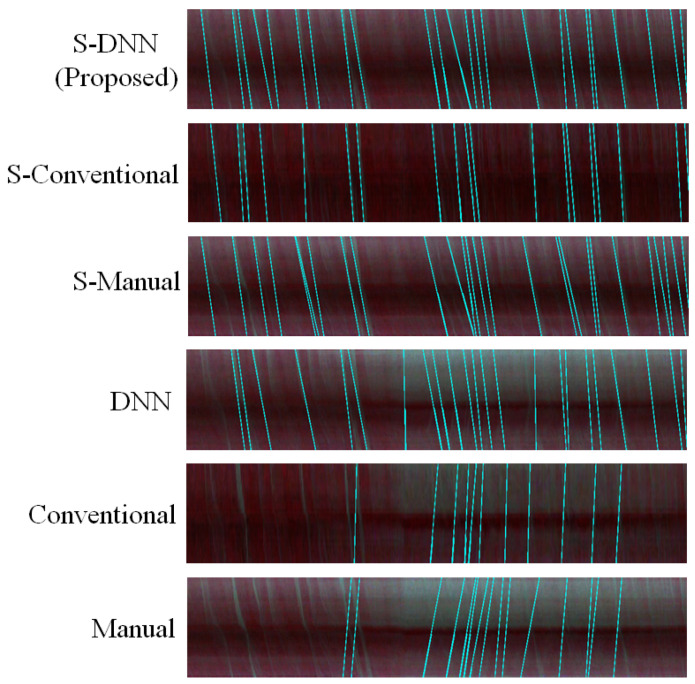
Events reconstructed from event counting using various methods for capillary 3.

**Figure A4:**
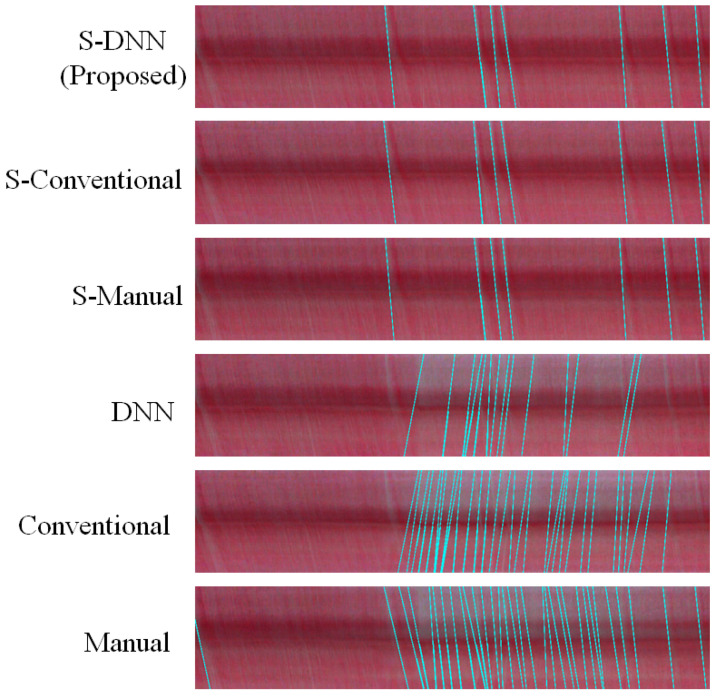
Events reconstructed from event counting using various methods for capillary 4 (same as Figure 8).

**Figure A5:**
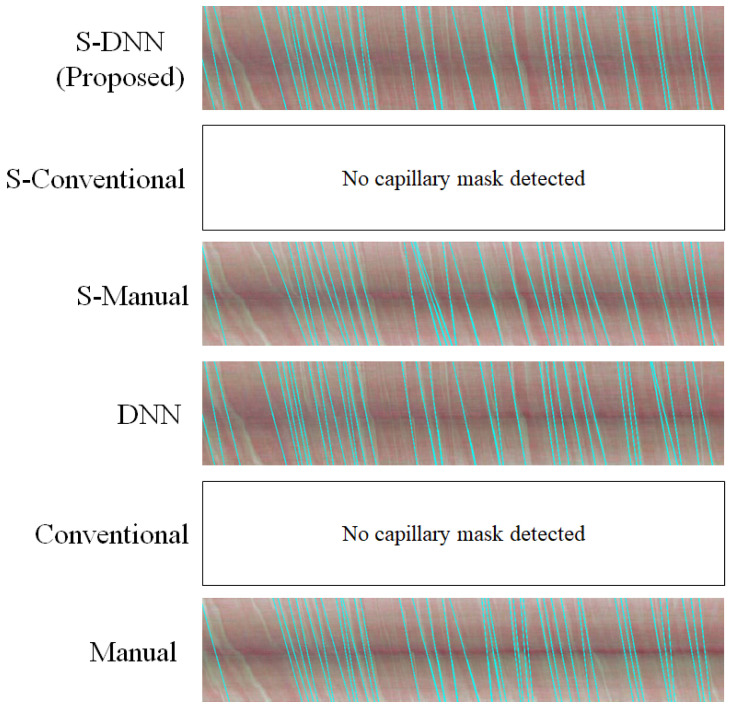
Events reconstructed from event counting using various methods for capillary 5.

**Figure A6:**
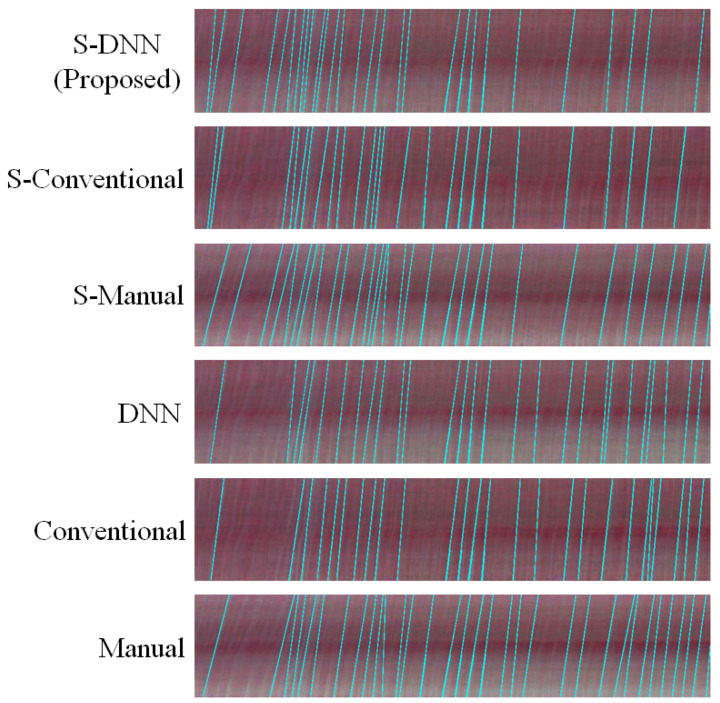
Events reconstructed from event counting using various methods for capillary 6.

In addition to the number of predicted events, Figure 8 shows that the proposed method correctly predicts the locations of events. In other words, a method may predict events in incorrect positions from an ST map, as shown in the results obtained from other methods. If a method predicts some events in incorrect positions, and, despite the number of predictions corresponding to the ground truth, the method could not ensure success for other input images. The proposed method captures all the lines in an ST map with correct position and number, further verifying its detection accuracy. The reconstructed lines from the ST maps of all the evaluated videos are given in Figure A1, Figure A2, Figure A3, Figure A4, Figure A5 and Figure A6 in Appendix B.

## 5. Discussion

### 5.1. Main Contributions

In this paper, we propose an automated method for detecting and counting WBCs that appear as visual gaps in nailfold capillary images. The proposed method consists of an automatic capillary segmentation method using deep learning, video stabilization, and WBC event detection algorithms. There has been no specific previous research on the WBC event detection using the nailfold capillary images. The most similar work has been conducted by Trinidad et al. [34]. However, our work has two major differences from theirs. While our work implements the automatic segmentation method and exploits only capillary-relevant intensity information, they used bounding box and brightness variations which could affect WBC count performance with noises in the background of capillary videos [34]. On the other hand, as the proposed method exploits intensities only in capillaries, it has less chance to be affected by noises in the background and therefore would be robust to the background noise. Since there is no specific previous work, the performances of the three segmentation algorithms (manual, conventional, and deep learning) with and without video stabilization were compared in this study. Experimental results demonstrate that the proposed method improves the performance of the WBC event counting and outperforms the conventional approach.

### 5.2. Segmentation Method

**Figure 9:**
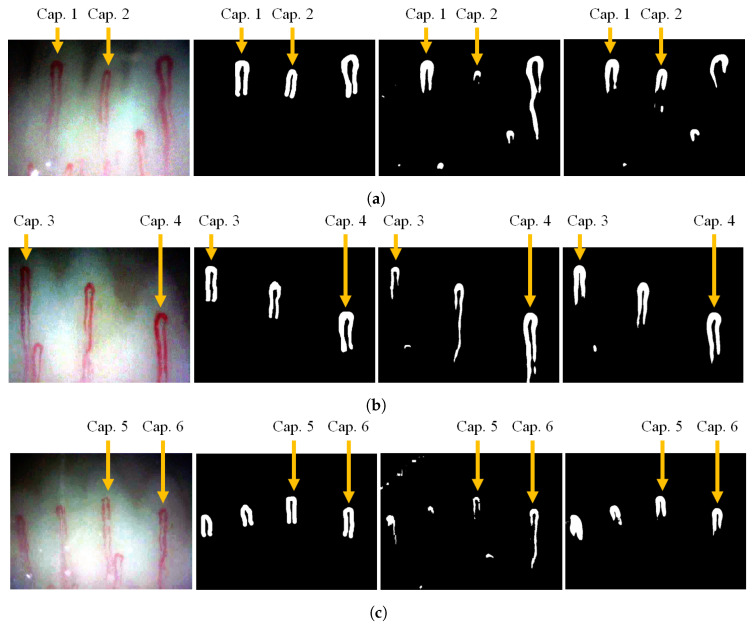
Capillary segmentation for videos (**a**) 1, (**b**) 2, and (**c**) 3. From left to right: the raw frame, manual segmentation, conventional segmentation, and (deep learning) semantic segmentation are displayed. The selected capillaries are indicated by the yellow arrows.

The semantic segmentation method uses a deep learning approach that could contribute to the automation of WBC event counting while replacing manual capillary segmentation. From left to the right in Figure 9, video frames and their corresponding capillary labels estimated using the manual, conventional, and deep learning segmentation are displayed.

**Figure 10:**
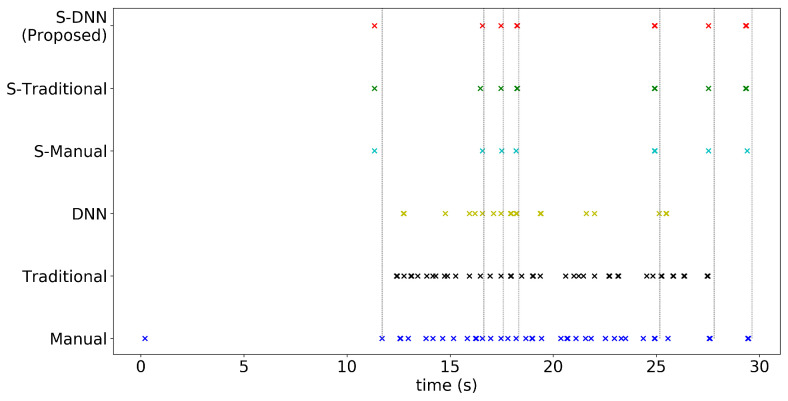
Predicted WBC event counts and ground truths for capillary 4. The *x*-axis denotes time stamp in the video, and the *y*-axis represents the different combinations of the video stabilization process and capillary segmentation methods. The gray vertical dashed lines indicate the events identified by experts. Note that all the segmentation methods with the stabilization preprocessing correctly recognize the counts of WBCs.

**Figure A7:**
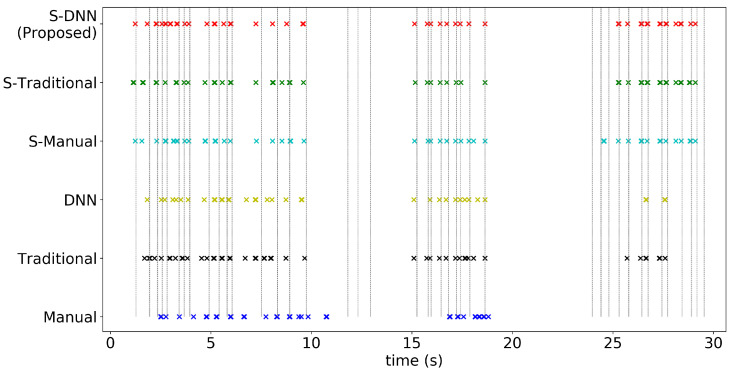
Prediction consistency of event counting compared with ground truths for capillary 1.

**Figure A8:**
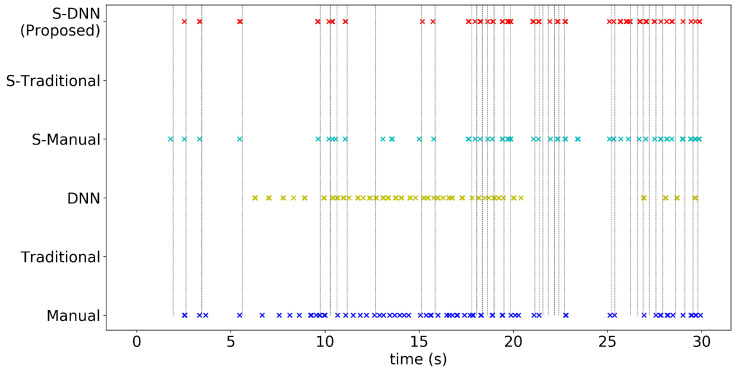
Prediction consistency of event counting compared with ground truths for capillary 2.

**Figure A9:**
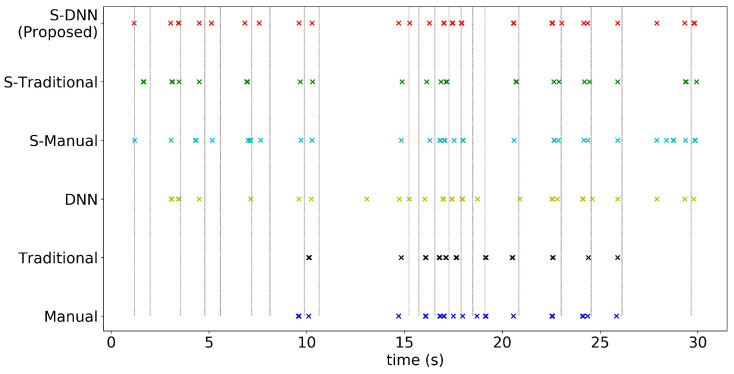
Prediction consistency of event counting compared with ground truths for capillary 3.

**Figure A10:**
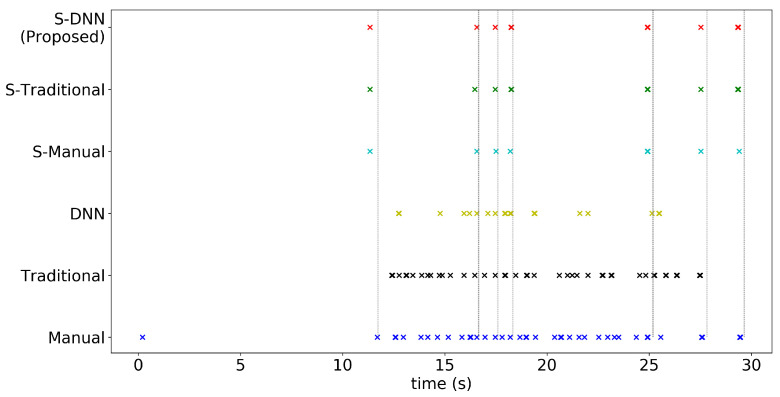
Prediction consistency of event counting compared with ground truths for capillary 4 (same as Figure 10).

**Figure A11:**
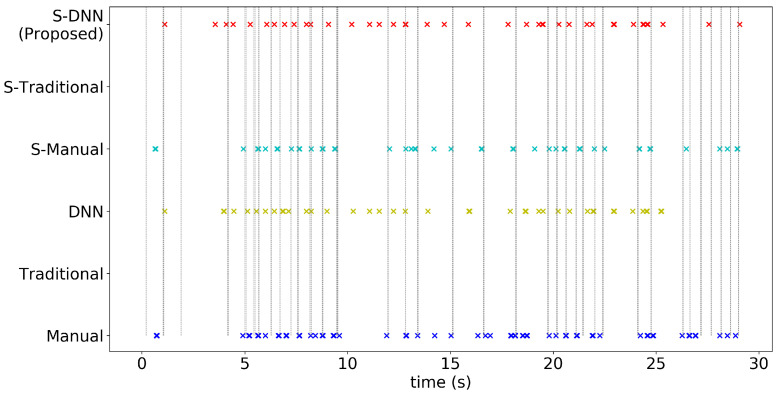
Prediction consistency of event counting compared with ground truths for capillary 5.

**Figure A12:**
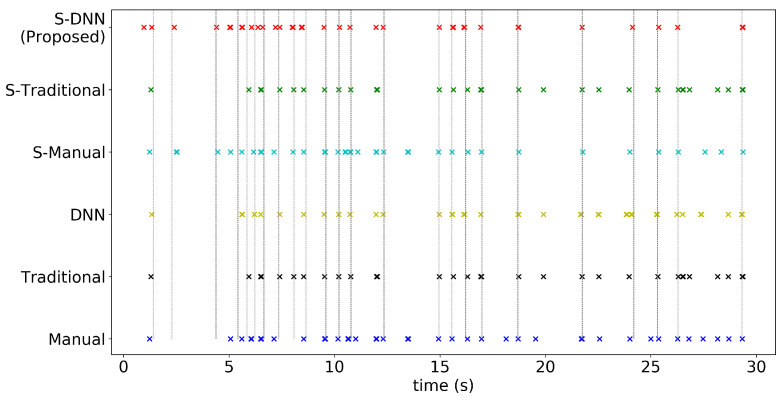
Prediction consistency of event counting compared with ground truths for capillary 6.

The labels obtained by the experts and using the deep learning are similar. Likewise, methods ‘S-DNN’ and ‘S-Manual’ in Figure 10 and the Figure A7, Figure A8, Figure A9, Figure A10, Figure A11 and Figure A12 in Appendix A show that labeling using the deep learning approach is close to that obtained manually. Therefore, event counting based on the deep learning has comparable performance to that based on the manual segmentation.

Furthermore, the deep learning approach is more reliable compared with the conventional approach for the automatic capillary segmentation. Figure 9 shows that the conventional segmentation is relatively sensitive to the image quality, whereas deep learning-based segmentation is more robust. As a matter of fact, some capillaries are not reflected in the conventional labels, as shown in Figure A1, Figure A2, Figure A3, Figure A4, Figure A5 and Figure A6 in Appendix B. Therefore, deep learning-based segmentation is robust to an image in low quality and outperforms the conventional segmentation approach.

### 5.3. Video Stabilization

To measure how much the video stabilization process affects the WBC event counting process, the results of the WBC event counting with and without the video stabilization were compared. Figure 10 shows that the methods with video stabilization correctly predict the seven ground-truth events, whereas those without video stabilization miss some of them and identify incorrect events. This is because the ST map from a video without stabilization captures the intensities of the background rather than those of a capillary, which appear as white areas in the ST map, and they are subsequently represented as peaks in the corresponding polar map. As the intensities of the background and events are almost the same without stabilization and both represent peaks in the polar map, event detection (Section 3.6) is degraded, resulting in the miscounts.

**Figure 11:**
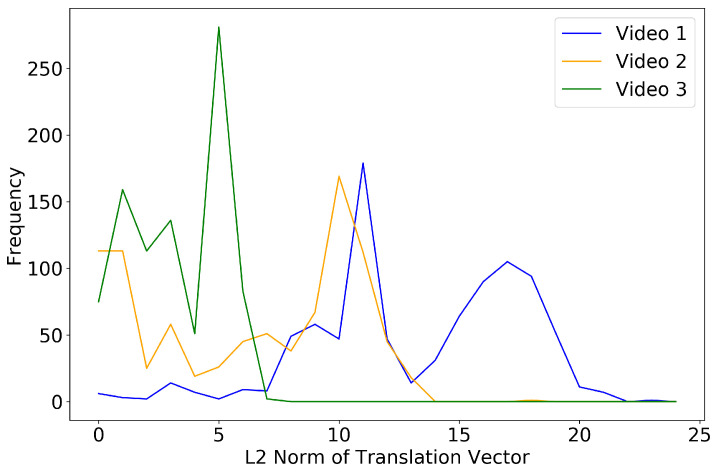
The distribution of the norms of translation vectors for three capillary videos. Each *x*- and *y*-axis indicates the L2 norm of a translation vector and the occurrence frequency of each norm.

Moreover, the norms of all translation vectors from the video stabilization process were acquired for each capillary video to measure the amount of the stabilization. If a capillary video contains more unwanted motion artifacts, then the overall norm of the translation vectors would become large and therefore the event counting results with and without the video stabilization process would differ a lot. Conversely, if a capillary video contains less motion artifacts, then the results would not differ that much. Figure 11 shows the distribution of the norms of translation vectors for each video, which could infer that videos 1 and 2 are more intensely stabilized than video 3 due to their bigger means of the L2 norm distribution. The Figure A7, Figure A8, Figure A9, Figure A10, Figure A11 and Figure A12 in Appendix A demonstrate that the predictions for capillaries 1–4 in videos 1 and 2 with all different stabilization/segmentation methods considerably differ from those for capillaries 5 and 6 in video 3.

Figure 8 and Figure 10 show the consistency between the proposed method and experts’ evaluation regarding the WBC event detection. In Figure 10, the gray vertical dashed lines and colored crosses indicate the events counted by an experts and those counted using the various methods, respectively. The cyan lines in Figure 8 visualize the counted events. The method with stabilization consistently predicts the events when compared with other methods. The event detection for all videos are reported in Figure A7, Figure A8, Figure A9, Figure A10, Figure A11 and Figure A12 in Appendix A, whose figures demonstrate the effectiveness of the video stabilization process on the prediction consistency of the event counting compared with the ground truths.

## 6. Conclusions

In this paper, a fully automated WBC event counting method is proposed to determine the number of visual gaps representing WBCs on capillary images using deep learning and video stabilization approaches. The proposed method segments capillary labels using a deep learning model and stabilizes the video frames with respect to the capillary labels to improve the performance of the event counting. The labels determined by the deep-learning-based segmentation are more reliable than those determined by the conventional segmentation, as the deep-learning-based segmentation gives labels close to ones obtained manually and the conventional segmentation fails to label capillaries in the case of low quality of images. Moreover, the video stabilization aligns capillaries to the same position to remove unwanted motion artifacts that might cause miscounts of events and therefore could guarantee accurate WBC event counting, resulting in comparable performance with the human experts.

## Figures and Tables

**Table 1:** Method configuration.

No.	Method	Segmentation Method	Video Stabilization
1	Manual	Manual	No
2	Conventional	Conventional	No
3	DNN	DNN	No
4	S-Manual	Manual	Yes
5	S-Conventional	Conventional	Yes
6	S-DNN	DNN	Yes

## Appendix A. Consistency of WBC Event Counting for Various Methods

The figures in this appendix show the ST maps and corresponding reconstructed events. The cyan lines are the reconstructed events after event counting using various methods. The abbreviations denoting method combinations are detailed in Section 4.

## Appendix B. Spatiotemporal Maps of Capillaries for Various Methods

The figures in this appendix show the prediction consistency of WBC event counting using various methods with respect to the ground truths provided by experts. The gray vertical dashed lines and colored crosses indicate events counted by experts and were obtained from the various methods, respectively. The abbreviations denoting method combinations are detailed in Section 4.
